# The role of metabolic health in neurostructural and cognitive alterations in bipolar disorders

**DOI:** 10.1017/S0033291726104905

**Published:** 2026-06-18

**Authors:** Maya Selitser, Sean R. McWhinney, Linzhi Wu, Julia Fraiha-Pegado, Lorielle Dietze, Emily Corkum, Lena Selitser, Abraham Nunes, Martin Alda, Katja Franke, Tomas Hajek

**Affiliations:** 1 Department of Psychiatry, Dalhousie University, Halifax, Canada; 2 Adler School of Professional Psychology: Adler University, Canada; 3 Jena University Hospital: Universitatsklinikum Jena, Germany

**Keywords:** bipolar disorders, BrainAGE, brain structure, cognition, metabolic syndrome, obesity

## Abstract

**Background:**

Bipolar disorders (BD) rank among the most disabling conditions, affecting millions worldwide. Cognitive impairment in BD is linked with brain changes and worse functional outcomes. Obesity and metabolic syndrome (MetSy) are overrepresented in BD and are associated with more pronounced brain structural and cognitive alterations in the general population. Here, we studied for the first time the contribution of metabolic dysfunction to both brain and cognitive alterations in BD.

**Methods:**

We recruited 163 participants (76 individuals with BD, 87 controls). We used principal component analysis (PCA) to derive a composite measure of metabolic health from WHR, BMI, HOMA-IR, HbA1c, TGC, HDL, LDL, systolic, diastolic BP, and to derive a composite cognitive measure from the California Verbal Learning Test and Digit Span. Brain structure was indexed using machine-learning-predicted BrainAGE derived from T1-weighted MRI.

**Results:**

Obesity, hypertension, insulin resistance, and dyslipidemia contributed most strongly to variance in metabolic health in this sample. This MetSy-associated risk cluster predicted higher BrainAGE (*β* = 0.75 ± 0.29, *p* = 0.011) and lower cognitive performance (*β* = −0.19 ± 0.09, *p* = 0.037), accounting for 30% of the association between BD and higher BrainAGE and 25% of the association between BD and worse cognitive performance. These effects were independent of symptoms, medications, illness course, and duration.

**Conclusions:**

MetSy, particularly obesity, was closely linked with brain/cognitive impairments in BD. While the need for metabolic monitoring should be informed by the diagnosis of BD, screening for MetSy can also help track brain health and cognitive functioning.

## Introduction

Due to early onset and lifelong course, bipolar disorders (BD) rank among the costliest medical conditions. Approximately 79% of the economic burden of BD is due to indirect costs such as occupational impairment, as opposed to direct treatment costs such as hospitalization (Gitlin & Miklowitz, 2017). Cognitive deficits, specifically in verbal learning and memory, attention, working memory, and executive function (Burdick et al., 2014; Huang et al., 2023; Jensen et al., 2016; Kurtz & Gerraty, 2009; Roux et al., 2017), persist into remission and are among the strongest predictors of psychosocial and occupational impairment in BD, even more so than clinical factors (Bonnín et al., 2010; Levy & Manove, 2012; Martinez-Aran et al., 2007). Brain structural alterations in BD, such as widespread grey matter loss and reduced hippocampal volumes, in turn may be associated with cognitive impairments (Moorhead et al., 2007; Yucel et al., 2007). Importantly, current first-line treatments such as antipsychotics or anticonvulsants do not adequately address these alterations and may even themselves contribute to poorer neurostructural (Abé et al., 2022; Hibar et al., 2016, 2018) and cognitive (Allott et al., 2023, 2024; Feber et al., 202 5; Husa et al., 2014) outcomes. Even though this is a key clinical concern, we do not understand the marked heterogeneity in cognitive and brain outcomes among people with the same diagnosis of BD, with some individuals demonstrating marked brain and cognitive alterations, while others do not (Angelescu et al., 2021; Burdick & Millett, 2021; Millett & Burdick, 2021; Wolfers et al., 2021). It thus becomes critical to attempt to identify extra-diagnostic variables which could explain part of this heterogeneity. Obesity and metabolic syndrome (MetSy) are key candidates here as they are overrepresented in BD and are associated with brain and cognitive outcomes.

Obesity is often comorbid with other cardiovascular risk factors, which together form the so-called metabolic syndrome. MetSy is defined by the presence of at least three of the following: obesity, low HDL, high triglycerides (TGC), insulin resistance, hyperglycemia, and hypertension (Meldrum et al., 2017). People with BD carry a 2.9-fold greater risk of obesity (Afzal et al., 2021), greater rates of individual cardiovascular risk factors, including elevated blood pressure, impaired fasting glucose, low HDL cholesterol, hypertriglyceridemia, and insulin resistance (Coello et al., 2019; Jirakran et al., 2025) and 3.5-fold greater risk of MetSy (Coello et al., 2019), compared to psychiatrically unaffected individuals. Obesity and MetSy in the general population are associated with brain and cognitive changes (Gómez-Apo et al., 2021; Raji et al., 2010; Selitser et al., 202 5; Vainik et al., 2013; Yang et al., 2018) often in the same brain regions or cognitive domains as those associated with BD, including ventricular enlargement, cortical thinning (McWhinney et al., 2021, 2022, 2023) and impairments in global cognition (Maksyutynska et al., 2024), attention, executive function, verbal and visual memory (Bora et al., 2019; Maksyutynska et al., 2024; Salvi et al., 2020). It is therefore unsurprising that among individuals with BD, those with versus without comorbid obesity/MetSy, abnormal blood lipid levels, or high blood pressure show more pronounced brain structural (Bora et al., 2019; Kennedy et al., 2021, 2022; McWhinney et al., 2021, 2023) and cognitive impairments (Depp et al., 2014; Naiberg et al., 2016a; Yim et al., 2012). Yet, there are no studies investigating the interplay between MetSy and both cognitive and brain changes in BD.

The fact that cardiometabolic risk factors do not exist in isolation and tend to cluster into MetSy creates analytical challenges. Within MetSy, cardiometabolic risk factors do not operate as independent predictors but as a correlated system arising from shared biological mechanisms. Consequently, analytical strategies that model these variables individually risk misattributing effects and underestimating their joint impact. Multivariate techniques, such as PCA, allow us to derive a composite metabolic score that captures shared variance across related measures and is thus closer to the multifactorial architecture of the cardiovascular risk.

To address these considerations, we collected brain scans, measures of MetSy, and multiple measures of cognition in a large sample of individuals with BD. To account for the complexity of these variables, we used multivariate approaches to generate composite measures of metabolic, cognitive, and brain health. We hypothesized that BD would be associated with higher BrainAGE, lower cognitive performance, and poorer metabolic health, with the latter partially mediating the effects of BD on brain structure and cognition.

## Methods

### Participant recruitment

We recruited participants with BD from a specialized tertiary care clinic, Mood Disorders Program at Dalhousie University, Halifax, NS, and community-dwelling healthy controls. Diagnostic interviews were performed by psychiatrists, according to the Schedule for Affective Disorders and Schizophrenia, Lifetime version (SADS-L), and diagnoses were made according to DSM-IV criteria. Included patients were required to (1) have the diagnosis of bipolar I or II disorder made by a psychiatrist, and (2) be at least 18 years of age. Patients were excluded if they had (1) the diagnosis of organic mood disorder; (2) mood disorder not otherwise specified; or (3) more than one lifetime course of electroconvulsive therapy or electroconvulsive therapy within the last 6 months. Controls were excluded if they had (1) a personal history of psychiatric disorders. Additional exclusion criteria for both groups included a history of (1) substance use disorder in the last 12 months; (2) neurological or cerebrovascular disorders, and any MRI contraindications.

### MRI acquisition

All magnetic resonance acquisitions were performed with a 1.5 Tesla General Electric Signa scanner (General Electric Medical Systems, Fairfield, Connecticut) and a standard quadrature head coil. After a localizer scan, a T1-weighted spoiled gradient recalled scan was performed (flip angle = 40°, echo time = 5 ms, repetition time = 25 ms, field of view = 24 cm × 18 cm, matrix = 256 × 160 pixels, number of excitations = 1, no interslice gap, 124 slices, 1.5 mm thick).

### BrainAGE estimation

We estimated the brain age of each participant using a machine learning method developed by us (K.F.), extensively validated (Franke et al., 2010, 2012), shown to be sensitive to metabolic or psychiatric disorders (Franke et al., 2013; Gaser et al., 2013; Hajek et al., 2019; Kolenic et al., 2018), and robust to inter-scanner differences (Franke et al., 2010, 2012). Briefly, this included (1) standard voxel-based morphometry preprocessing of structural MRI data, (2) feature reduction via smoothing and PCA, and (3) age estimation using relevance vector regression (RVR). This RVR model was trained using an independent sample of 504 healthy individuals from the IXI database (http://www.brain-development.org). For more details, see (Franke et al., 2010, 2013; Gaser et al., 2013). We calculated the brain age gap estimate (BrainAGE) as the difference between each individual’s chronological age and predicted brain age. The mean absolute error (MAE) was 6.34 years among healthy test subjects, with an excellent agreement between chronological age and brain age (intraclass correlation [ICC] = 0.85, 95% confidence interval [CI] = [0.78, 0.90]. This is comparable to our previous validation of the method (MAE = 5.08, *R*^2^ = 0.83) (Franke et al., 2010) and to other models, that is, MAE = 4.6 years, *R^2^* = 0.83 (Koutsouleris et al., 2014), and MAE = 4.31, *R*^2^ = 0.79 (Schnack et al., 2016).

### Cognitive data

Verbal learning and memory was assessed using the second edition of the California Verbal Learning Test (CVLT-II) (Woods et al., 2006), from which we included the following measures: (1) the sum of recalled words across Trials I-V; (2) learning slope (i.e., the difference between each participant’s Trial V and Trial I scores, adjusted for Trial I score), (3) Short-Delay free recall scores, (4) Long-Delay free recall scores, Total (5) intrusion and (6) repetition errors. We further assessed executive function and cognitive flexibility by deriving two performance contrast measures, as per the CVLT-II scoring manual: (1) proactive interference, and (2) retroactive interference scores. Negative scores represent greater retroactive/proactive interference.

Auditory attention was assessed using the forward digit span test, and working memory was assessed using the backward digit span test of the Wechsler Adult Intelligence Scale III (WAIS-III) (Wechsler, 2019).

### Other variables

Prior to scanning, we collected detailed personal history focusing on hypertension, diabetes mellitus, myocardial infarction, and other somatic comorbidities as well as medication history. Full psychiatric evaluations included information regarding BD type, age of onset, number of prior hospitalizations, number of manic and/or depressive episodes, duration of illness, and current medications.

On the day of scanning, we obtained symptoms ratings using the Young Mania Ratings Scale (YMRS) (Young et al., 1978) and Hamilton Depression (HAM-D) scale (Kobak, 2004). We collected anthropometric measures, including weight, height, hip, and waist circumferences. Body mass index (BMI) was calculated using the formula: BMI = weight (kg)/height (meters)^2^. Waist-to-hip ratio (WHR) was calculated by dividing waist circumference by hip circumference. We collected fasting blood samples, and an assessment of blood metabolite levels was performed in a single clinical laboratory using standard methods. We measured LDL-cholesterol, HDL-cholesterol, fasting TGC, high-sensitivity C-reactive protein (CRP), fasting glucose, glycosylated hemoglobin (HbA1c), and fasting insulin. We calculated the homeostatic model assessment of insulin resistance (HOMA-IR) using the equation,

HOMA-IR=[fasting plasma insulin(mU/L)×fasting plasma glucose(mmol/L)]/22.5.

### Principal component analysis

We used PCA to derive a composite measure of MetSy from the individual components, including: (1) WHR, (2) BMI, (3) HOMA-IR, (4) HbA1c, (5) TGC, (6) HDL, (7) LDL, (8) Systolic BP, (9) Diastolic BP. To uphold the construct validity of Metabolic PC1 as a latent measure of MetSy, we did not include CRP and tested it separately as a mediator. Missing values (maximum of 12.27% in WHR) were imputed using the missForest algorithm, using the R package *missForest* (Stekhoven & Bühlmann, 2012). The PC1 scores for each individual were extracted and used as a continuous variable (Metabolic PC1) in subsequent analyses.

Similarly, we used PCA to derive a composite measure of cognitive function from the following cognitive test scores: (1) Sum of Trials I-V), (2) learning slope, (3) Short-Delay free recall, (4) Long-Delay free recall, (5) total intrusions, (6) total repetitions, (7) retroactive interference, (8) proactive interference, (9) Forward digit span, (10) Backward digit span. Missing values (maximum of 20.25% for learning slope, retroactive interference, and proactive interference) were imputed using *missForest* (Stekhoven & Bühlmann, 2012). The PC1 scores for each individual were extracted and used as a continuous variable (Cognitive PC1) in subsequent analyses. All variables were standardized (z-scored) prior to PCA to ensure comparability across different scales. PCA was performed using the *prcomp* function in R.

### Statistical analyses

We compared demographic and clinical characteristics, as well as BrainAGE and cognitive test scores, between BD and controls using multiple linear regression for continuous variables and logistic regression for categorical variables, with age and sex as covariates. Measures that were not normally distributed were transformed using a Box-Cox transformation, which included TGC, HDL, fasting glucose, HbA1c, HOMA-IR, and CRP. Total repetitions and total intrusions were transformed using the Yeo-Johnson transformation (Yeo & Johnson, 2000), as this best improved normality, using the R package *bestNormalize.*

We first tested associations between metabolic health, BrainAGE, and cognitive performance. We created linear regression models to test Metabolic PC1 as a predictor of either BrainAGE or Cognitive PC1. To test whether brain structural changes were associated with cognitive alterations, we tested whether BrainAGE significantly predicted Cognitive PC1. All models controlled for age, sex, and diagnosis. Lithium treatment was additionally included as a covariate in all models where BrainAGE served as the outcome, to account for its well-established neuroprotective effects (Forlenza et al., 2014; Van Gestel et al., 2019).

We next tested whether metabolic health was associated with brain structural differences in BD. To this goal, we ran a mediation model with diagnosis as the independent variable, BrainAGE as the dependent variable, and Metabolic PC1 as the mediator. We also tested whether metabolic health statistically predicted cognitive alterations in BD. To this goal, we performed a mediation analysis with diagnosis as the independent variable, Cognitive PC1 as the dependent variable, and Metabolic PC1 as the mediator. Lastly, we tested whether CRP mediated the association between metabolic PC and brain or cognitive measures. All mediation analyses controlled for age and sex, and included average direct effects (ADE) and average causal mediated effects (ACME) using a quasi-Bayesian approximation of the 95% confidence interval (95% CI) using the R package *mediation* (Tingley et al., 2014) with 1,000 bootstrapped simulations.

To test for possible confounding effects of symptoms and medications, we used linear regression in participants with BD to test whether Metabolic PC1 or BrainAGE or Cognitive PC1 were predicted by YMRS and HAM-D scores, number of prior episodes, number of prior hospitalizations, duration of illness, use of antipsychotics, antidepressants, anticonvulsants, or benzodiazepines, adjusting for age and sex. Any clinical variables that were significantly associated with a given outcome of interest were then tested in the same model.

## Results

### Sample descriptives

The sample consisted of 163 participants, including 76 BD (46.6%) and 87 control individuals. The mean age of participants was 43.14 (±15.26). Relative to controls, participants with BD were older, had lower concentrations of HDL, higher concentrations of TGC, greater BMI, waist circumference, and WHR. They were comparable to controls on digit span scores but performed worse on all measures of verbal learning and memory (see Tables 1 and 2).

**Table 1.** Participant demographic characteristics are presented by group, with significance indicated as follows: *p < 0.05, **p < 0.01, ***p < 0.001 Table 1. long description.

	Control	Patient	Significance of Group Difference
*n*	87	76	
Age (mean (SD))	39.88 (14.68)	46.82 (15.15)	*F* (1,159) = 8.76, *p* = 0.004**
Sex (% Male)	34 (39.1)	34 (44.7)	*F* (1,159) = 0.5, *p* = 0.479
Diabetes (% Yes)	8 (9.8)	13 (21.0)	*F* (1,139) = 1.41, *p* = 0.238
Hypertension (% Yes)	13 (14.9)	22 (28.9)	*F* (1,158) = 1.49, *p* = 0.225
Overweight/Obese, BMI > 25 kg/m^2^ (% Yes)	48 (55.2)	59 (77.6)	*F* (1,158) = 5.78, *p* = 0.017*
TGC, mmol/L (mean (SD))	1.11 (0.66)	1.61 (1.57)	*F* (1,150) = 8.05, *p* = 0.005**
LDL, mmol/L (mean (SD))	2.72 (0.78)	2.82 (0.81)	*F* (1,150) = 0.04, *p* = 0.836
HDL, mmol/L (mean (SD))	1.50 (0.46)	1.26 (0.34)	*F* (1,150) = 16.05, *p* < 0.001***
Fasting glucose, mmol/L (mean (SD))	5.13 (1.44)	5.18 (1.10)	*F* (1,149) = 0, *p* = 0.955
HbA1c (mean (SD))	5.31 (0.80)	5.58 (2.65)	*F* (1,134) = 1.83, *p* = 0.179
HOMA-IR (mean (SD))	2.06 (1.45)	2.50 (2.15)	*F* (1,146) = 0.82, *p* = 0.367
CRP, mg/mL (mean (SD))	3.08 (4.62)	6.63 (12.55)	*F* (1,136) = 2.11, *p* = 0.148
Systolic blood pressure (mean (SD))	115.15 (12.84)	120.54 (13.42)	*F* (1,151) = 2.25, *p* = 0.136
Diastolic blood pressure (mean (SD))	75.08 (10.21)	77.90 (12.46)	*F* (1,151) = 1.72, *p* = 0.192
BMI (mean (SD))	26.12 (4.88)	30.26 (6.21)	*F* (1,158) = 18.25, *p* < 0.001***
Waist circumference, cm (mean (SD))	88.32 (14.92)	101.57 (15.57)	*F* (1,154) = 20.8, *p* < 0.001***
Waist-to-hip ratio (mean (SD))	0.86 (0.09)	0.92 (0.08)	*F* (1,137) = 14.7, *p* < 0.001***

*Note:* All comparisons are adjusted for age and sex.

**Table 2.** Participant cognitive performance presented by group, with significance indicated as follows: *p < 0.05, **p < 0.01, ***p < 0.001 Table 2. long description.

	Control	Patient	Significance of group difference
*N*	87	76	
CVLT trial I (mean (SD))	7.24 (2.10)	6.61 (2.21)	*F* (1,120) = 1.04, *p* = 0.309
CVLT trial V (mean (SD))	13.52 (2.10)	12.07 (2.72)	*F* (1,120) = 6.13, *p* = 0.015*
Sum trials I-V (mean (SD))	54.77 (10.76)	48.58 (11.43)	*F* (1,158) = 7.64, *p* = 0.006**
List B (mean (SD))	6.32 (2.08)	5.14 (1.96)	*F* (1,158) = 8.36, *p* = 0.004**
Short delay free recall (mean (SD))	11.90 (3.00)	9.97 (3.47)	*F* (1,158) = 8.34, *p* = 0.004**
Long delay free recall (mean (SD))	12.24 (3.13)	10.51 (3.63)	*F* (1,156) = 6.44, *p* = 0.012*
Total intrusions (mean (SD))	2.06 (2.59)	2.76 (3.50)	*F* (1,158) = 0.11, *p* = 0.739
Total repetitions (mean (SD))	3.36 (3.26)	2.38 (2.50)	*F* (1,158) = 2.75, *p* = 0.099
Learning slope (mean (SD))	6.27 (1.96)	5.46 (2.42)	*F* (1,119) = 5.09, *p* = 0.026*
Retroactive interference (mean (SD))	−1.76 (1.87)	−1.80 (2.06)	*F* (1,120) = 0.12, *p* = 0.725
Proactive interference (mean (SD))	−0.65 (2.12)	−1.15 (2.22)	*F* (1,120) = 1.5, *p* = 0.223
Forward digit span (mean (SD))	11.01 (2.27)	11.10 (2.45)	*F* (1,145) = 0, *p* = 0.960
Backward digit span (mean (SD))	9.25 (2.46)	8.75 (2.36)	*F* (1,145) = 1.33, *p* = 0.252

*Note:* All comparisons are adjusted for age and sex, with additional control for CVLT Trial I score in Learning Slope analyses.

### Metabolic and cognitive principal components

**Figure 1.**
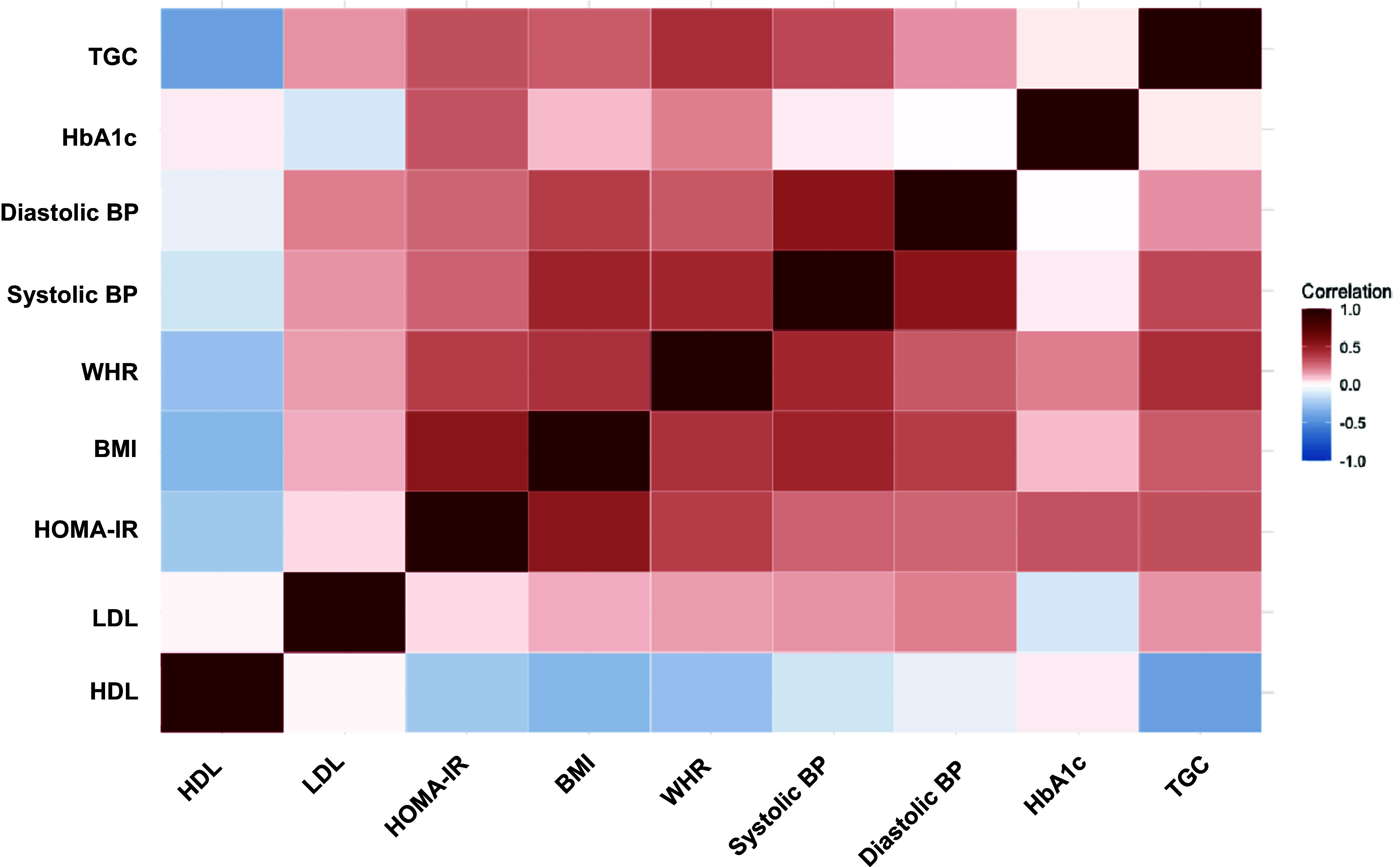
Correlation matrix showing relationships among the nine metabolic variables used in the metabolic principal component analysis. Figure 1. long description.

The first metabolic principal component (Metabolic PC1) accounted for 34.7% of variance across the nine metabolic/cardiovascular measures. Metabolic PC1 captured the MetSy constellation and showed the highest loadings for BMI (0.43) and WHR (0.41), followed by systolic BP (0.40) and HOMA-IR (0.38), see Figure 1 and Supplementary Table S1. Therefore, a higher metabolic PC1 score indicated higher (worse) metabolic scores, in proportion to the loadings.

The first cognitive principal component (Cognitive PC1) accounted for 34.4% of the variance across the 10 cognitive measures. Cognitive PC1 showed the highest loadings for Short-Delay free recall (0.51), Long-Delay free recall (0.49), and Sum of Trials I-V (0.46), see Supplementary Table S2, suggesting that it primarily represents verbal learning and memory. Higher cognitive PC1 scores, therefore indicated better performance.

### Associations between metabolic health, BrainAGE, and cognitive performance

**Figure 2.**
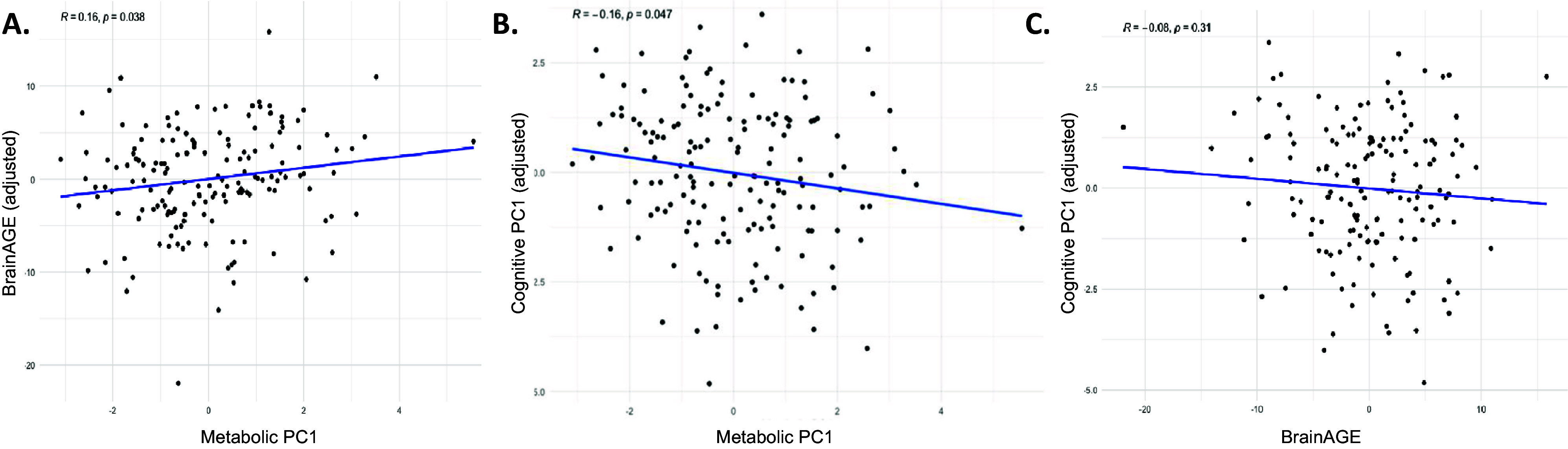
Partial effects adjusting for age, sex, diagnosis, and, in the case of BrainAGE, also lithium treatment. (a) Metabolic PC1 was significantly associated with BrainAGE (*r* = 0.16, *p* = 0.038) and (b) with Cognitive PC1 (*r* = −0.16, *p* = 0.047). (c) BrainAGE was not associated with Cognitive PC1 (*r* = −0.08, *p* = 0.310). Figure 2. long description.

In the overall sample, greater Metabolic PC1 scores significantly predicted higher BrainAGE (*β* = 0.75 ± 0.29, *t*(157) = 2.58, *p* = 0.011*, Figure 2a) and lower Cognitive PC1 scores (*β* = −0.19 *±* 0.09, *t*(157) = −2.11, *p* = 0.037*, Figure 2b), when controlling for age, sex, diagnosis and in case of BrainAGE, also Lithium treatment. BrainAGE did not predict Cognitive PC1 scores (*β* = −0.01 ± 0.02, *t*(157) = −0.46, *p* = 0.645, Figure 2c).

### Mediation analyses

**Figure 3.**
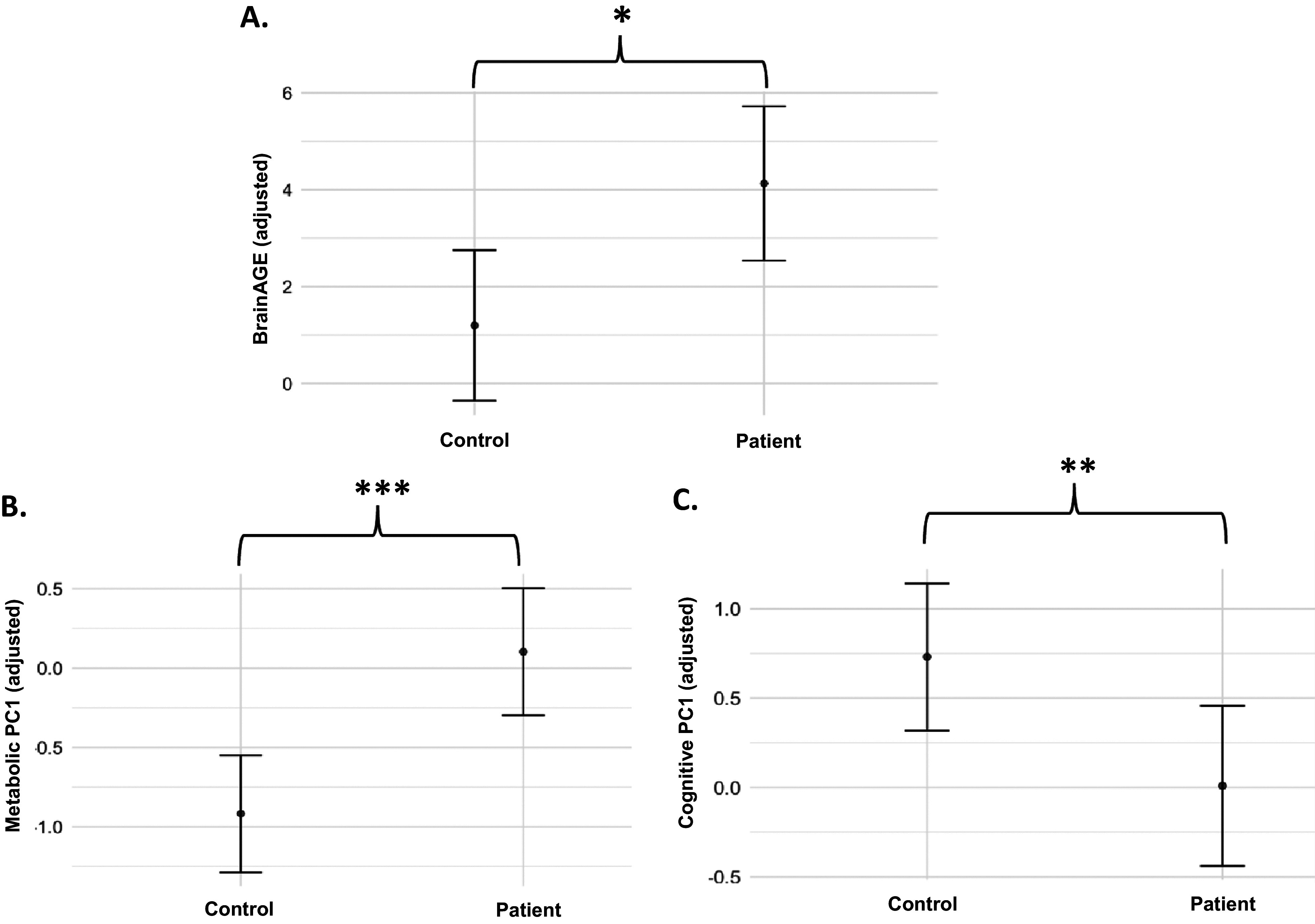
Adjusted mean metabolic and cognitive scores in patients and controls. The black center dots represent group means, adjusted for age and sex, and the bars represent their 95% confidence intervals. Compared to controls, patients had (a) significantly higher adjusted mean BrainAGE (F (1, 157) = 6.76, *p* = 0.010*), controlling for lithium treatment, (b) significantly higher (worse) Metabolic PC1 scores (*F*(1, 158) = 18.08*, p <* 0.001***) and (c) significantly lower (worse) Cognitive PC1 scores (*F*(1, 158) = 7.65*, p =* 0.006**). Figure 3. long description.

Individuals with BD had a significantly higher BrainAGE than controls, when adjusting for age, sex, and lithium treatment. Patients also had significantly higher Metabolic PC1 scores and lower Cognitive PC1 scores, adjusting for age and sex (Figure 3).

**Figure 4.**
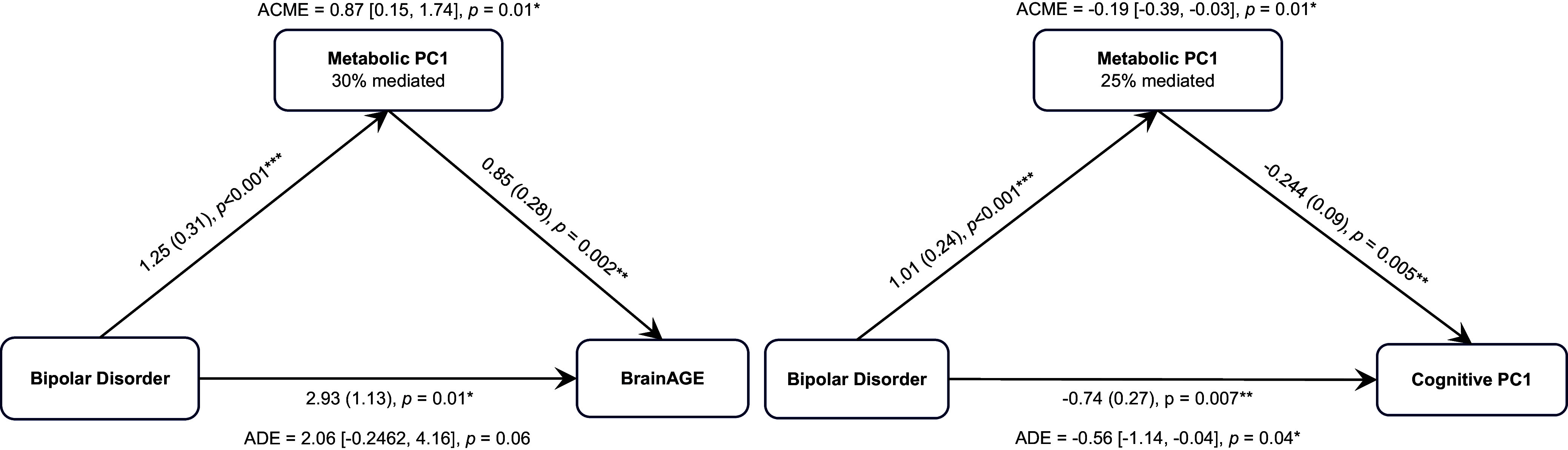
Results of the first mediation analysis, showing coefficients and their standard errors for associations between BD, Metabolic PC1, (a) BrainAGE and (b) cognition, while controlling for participant age, sex, and lithium treatment (where applicable), as well as the average causal mediated effect (ACME), and average direct effect (ADE). Figure 4. long description.

Metabolic PC1 scores significantly mediated 30% of the association between BD and BrainAGE (ACME = 0.87, 95%CI[0.15, 1.74], *p* = 0.014; Figure 4a), while controlling for age, sex, and lithium treatment, and 25% of the association between BD and Cognitive PC1 scores (ACME = −0.19, 95%CI[−0.39, −0.03], *p* = 0.012; Figure 4b), controlling for age and sex.

Higher serum CRP was significantly associated with Metabolic PC1 (*β* = 0.61 ± 0.09, *p* < 0.001), however it was not associated with either BrainAGE (*β* = 0.36 ± 0.40, *p* = 0.37) or Cognitive PC1 (*β* = 0.04 ± 0.12, *p* = 0.72), see Supplementary Table S3. Consequently, CRP did not significantly mediate the association between Metabolic PC1 and either BrainAGE (ACME = −0.05, 95%CI [−0.39,0.25], *p* = 0.692) or Cognitive PC1 (ACME = 0.07, 95%CI[−0.03,0.19], *p =* 0.162).

### Potential confounding by symptoms and medications

Out of all clinical factors (Table 3), only benzodiazepine use was associated with Metabolic PC1. However, it was not significantly associated with either BrainAGE or Cognitive PC1 and therefore cannot confound the associations between Metabolic PC1 and either outcome measure.

**Table 3.** Participant clinical characteristics are presented by group Table 3. long description.

	Controls	Patients
*N*	87	76
Age of onset (mean (SD))	NA	23.99 (9.30)
BD type (Type 2 (%))	NA	21 (27.6)
Number of prior episodes (mean (SD))	NA	10.35 (11.28)
Number of prior hospitalizations (mean (SD))	NA	2.22 (4.94)
Duration of illness, years (mean (SD))	NA	20.74 (12.00)
ADHD (Yes (%))	1 (1.1)	2 (2.6)
Anxiety disorders (Yes (%))	6 (6.9)	24 (31.6)
Eating disorders (Yes (%))	0 (0.0)	1 (1.3)
Substance use disorder (Yes (%))	1 (1.1)	12 (15.8)
Other psychiatric disorder (Yes (%))	1 (1.1)	9 (11.8)
Antipsychotics (Yes (%))	0 (0.0)	38 (50.0)
Anticonvulsants (Yes (%))	2 (2.3)	36 (47.4)
Antidepressants (Yes (%))	2 (2.3)	30 (39.5)
Benzodiazepine (Yes (%))	0 (0.0)	15 (19.7)
Lithium (Yes (%))	0 (0.0)	42 (55.3)
HAM-D score (mean (SD))	1.25 (1.97)	5.41 (5.44)
YMRS score (mean (SD))	0.60 (1.25)	1.92 (3.04)

When included in the same model with age and sex, both Metabolic PC1 and antipsychotics remained significant predictors of BrainAGE among patients (Metabolic PC1 *β* = 1.03 ± 0.35, *p* < 0.01; Antipsychotics *β* = 2.56 ± 1.08, *p* = 0.02, Supplementary Table S4). In the same model, lower Cognitive PC1 scores were predicted by treatment with antipsychotics ( *β* = −1.05 ± 0.40, *p* = 0.01) and duration of illness (= − 0.06 ± 0.02, *p* = 0.03), but not by Metabolic PC1 ( *β* = −0.10 ± 0.13, *p* = 0.47, Supplementary Table S5). Importantly, neither treatment with antipsychotics nor duration of illness was associated with Metabolic PC1 and therefore could not confound the association between Metabolic PC1 and brain or cognitive changes.

## Discussion

In this study, we showed that MetSy was associated with higher BrainAGE and worse cognitive functioning. Additionally, for the first time, we demonstrated that the brain and cognitive alterations cross-sectionally associated with BD were, in part, accounted for by poorer metabolic health. More specifically, 25–30% of the association between BD and cognitive or brain changes was related to the presence of MetSy.

Our results are in keeping with other studies showing that obesity/MetSy are associated with brain structure or cognition in people with severe mental illness (SMI). A series of studies have demonstrated lipid and blood pressure associations with brain structure or cognition in adolescents with bipolar disorder (Kennedy et al., 2021, 2022; Naiberg et al., 2016a). Our group has previously shown that in a young population (average age 28 years), with considerable prevalence of overweight/obesity (33.2%) but low rates of additional cardiovascular risk factors, central obesity alone mediated 15% of the association between first-episode psychosis (FEP) and higher BrainAGE (Kolenič et al., 2025). In the present study, with a higher average age ~ 43 years and consequently also higher rates of obesity and additional cardiovascular risk factors, MetSy explained a markedly higher proportion of the association between SMI and higher BrainAGE, that is, 30%. Taken together, these results suggest that individual components of MetSy not only relate to brain structure/cognitive functioning already early in the course of psychiatric illness, but they also add to the statistical effects of SMI, and cumulatively, their contributions become more pronounced in middle-aged individuals.

Our findings align with previous studies that link metabolic disturbances, including obesity, diabetes, and hypertension, to widespread reductions in cortical thickness, subcortical volumes (Feng et al., 2020; Gianaros et al., 2006; Li et al., 2023; Mahmood et al., 2025; Won et al., 2024), and higher BrainAGE (Selitser et al., 202 5). A recent study by Peterson et al. (2024) showed that greater severity of obesity, dyslipidemia, arterial hypertension, and insulin resistance was related to neurostructural alterations and that BMI, WHR, hip, and waist circumferences accounted for most of the shared variance in their multivariate predictor. This closely aligns with our results, showing that within MetSy, measures of obesity (i.e., BMI and WHR) had the highest loadings and were therefore the strongest contributors to a latent variable that was negatively associated with brain structure. Other studies have also demonstrated unique partial effects of obesity even when controlling for other cardiovascular risk factors (Busby et al., 2023; Selitser et al., 202 5). While MetSy will exert a greater effect on the brain than obesity alone, even within MetSy, obesity remains a key predictor of neuroanatomical impairments. Therefore, obesity should be a target for monitoring/intervention even in the absence of other cardiovascular risk factors.

Our findings further align with previous studies reporting associations between MetSy and cognitive impairments (Angoff et al., 2022; Cheon, 2023; Prickett et al., 2018; Takeuchi et al., 2012; Yang et al., 2018), including those involving attention (Koutsonida et al., 2022; Marseglia et al., 2021), verbal fluency (Marseglia et al., 2021), memory (Cavalieri et al., 2010; Hassenstab et al., 2010; Koutsonida et al., 2022), and executive function (Cavalieri et al., 2010; Hassenstab et al., 2010; Koutsonida et al., 2022; Marseglia et al., 2021; Schuur et al., 2010) in the general population or in BD (Aminoff et al., 2013; Bauer et al., 2015; Huang et al., 2023; Kennedy et al., 2022; Kurtz & Gerraty, 2009; Maksyutynska et al., 2024; Naiberg et al., 2016b; Piedrahíta Palacio et al., 2024; Poletti et al., 2015). In our study, MetSy partially accounted for cognitive changes in BD. More specifically, 25% of the association between BD and cognition was related to the presence of MetSy. This is worrisome considering the high rates of MetSy in BD (Antony et al., 2024), but it is also positive, as these results indicate that studies that did not control for MetSy likely inflated the extent of cognitive changes in BD. While we caution against interpreting such cross-sectional associations as causation, it would be worth testing whether treatment of MetSy-related changes could decrease cognitive impairment in BD.

We can only speculate about the mechanisms underlying the associations between MetSy and brain/cognitive changes. As ours is a cross-sectional study, we need to consider multiple directions of causation. It is possible that certain brain structural configurations predispose people to obesity/MetSy. If this were the case, we would expect to see more localized changes in regions underlying relevant cognitive/executive functions (impulsivity, reward dependence, homeostatic monitoring), rather than more global alterations, such as those captured by BrainAGE. We would also expect to see greater impairments in executive functioning, while our PCA was mostly capturing changes in verbal memory. It may also be that MetSy causes brain changes either directly or indirectly via additional lifestyle factors, that is, diet and exercise, which may be associated with both obesity and brain changes. In addition, both obesity and brain changes may be preceded by common genetic and/or developmental risk factors. While more than one explanation can apply, considering longitudinal studies (Fraiha-Pegado et al., 2026), the results of Mendelian randomization studies (Chen et al., 2023; Debette et al., 2014; Feng et al., 2023) and studies in which brain and cognitive changes were reversed following bariatric surgery (Duan et al., 2025; Legault et al., 2024; Tuulari et al., 2016; Vreeken et al., 2023; Zeighami et al., 2022), there is a growing body of evidence in support of obesity/MetSy not only preceding, but likely causing brain changes and cognitive alterations. Many mechanisms are invoked when speculating on the effects of obesity/MetSy on the brain, including pro-inflammatory signaling, oxidative stress, abnormal brain lipid metabolism, and impaired vascular reactivity, among others (Cai & Liu, 2012; Fernández-Sánchez et al., 2011; Ghowsi et al., 2021; Mattson, 2009). Interestingly, a marker of systemic inflammation (CRP), while associated with Metabolic PC1, was not associated with cognitive or brain changes and did not significantly mediate the association between Metabolic PC1 and either BrainAGE or Cognitive PC1.

In people with BD, we also need to consider the impact of obesogenic psychiatric medications, and that greater medication exposure could indicate a more severe illness, likely with more pronounced brain changes. However, the metabolic, brain, or cognitive outcomes were not associated with illness severity. Current treatment with antipsychotics significantly predicted higher BrainAGE in our sample, which replicated previous findings (Germaná et al., 2010; Hibar et al., 2016, 2018; Konopaske et al., 2008; Lieberman et al., 2003; Roiz-Santiañez et al., 2015; Voineskos et al., 2020). Obesogenic medications may be associated with brain changes via obesity, but the negative effect of antipsychotics persisted, along with a significant effect of Metabolic PC1. In contrast with this, in a single model, antipsychotic medications and duration of illness, but not Metabolic PC1, were significantly associated with cognitive PC1 in people with BD. Importantly, neither of these factors was significantly associated with Metabolic PC1, suggesting that while these illness-related factors may influence brain and cognitive outcomes in BD, their effects are independent of those exerted by poor metabolic health. This is further demonstrated by the fact that about half of the sample, in which we found the links between MetSy and cognitive or brain changes, were controls with no exposure to psychiatric medications and no diagnosis of BD.

Current guidelines recommend monitoring of metabolic markers in BD due to medical concerns. At the same time, if over a quarter of brain/cognitive changes in BD are associated with abnormal metabolic markers, clinicians may use the presence of MetSy as a marker of brain and cognitive alterations, reducing the need to perform costly MRIs or lengthy cognitive assessments. Furthermore, realizing that obesity and MetSy closely correlate with brain and cognitive health could be an important additional impetus for patients to adhere to lifestyle changes. Measuring obesity, which can be done in the office and does not require blood collection/lab analyses, is perhaps the easiest and yet most important component of monitoring for metabolic markers that predict brain and cognitive changes. While association does not mean causation, it would be worth testing whether these MetSy-related changes could be reversed by treatment, which would constitute a new option for treating brain/cognitive impairment in BD. In fact, studies of weight loss following bariatric surgery show significant, widespread increases in white and gray matter densities (Legault et al., 2024; Michaud et al., 2020; Rullmann et al., 2019; Tuulari et al., 2016), reduced BrainAGE (Zeighami et al., 2022), and improved performance across multiple cognitive domains (Duan et al., 2025; Vreeken et al., 2023).

This study also has important research implications. Metabolic markers should be controlled for in future studies to avoid inflating the effects of BD. Additionally, the contribution of metabolic markers to brain changes in BD complicates the use of brain scans to diagnose BD. Third, the varied presence of MetSy would contribute to a varying extent of brain/cognitive changes in BD, thus explaining some part of the neurostructural and cognitive heterogeneity. Lastly, perhaps some of the similarities in brain imaging changes between BD and schizophrenia reflect the shared presence of obesity/MetSy in both conditions, rather than any similarities/overlaps between illnesses.

A major strength of our study is the use of two data-driven approaches, BrainAGE and PCA, to reduce multiple complex and interrelated measures into a single composite score. The combination of these methods offers a statistically robust and objective framework to evaluate the complex interplay between metabolism, brain structure, and cognition in BD. Importantly, we focused on a highly prevalent yet understudied issue in the context of brain/cognitive changes, which are typically investigated separately, and included detailed clinical information obtained through regular follow-ups at a specialized mood disorders clinic, conducted by psychiatrists directly involved in the study. Our results are generalizable, as the prevalences of each metabolic condition in our sample mirrors that seen in the broader North American population, which is estimated as 73.6% for overweight/obesity (Centers for Disease Control and Prevention, 2024), 11.6% for diabetes (Centers for Disease Control and Prevention, 2025b), and 48.1% for hypertension (Centers for Disease Control and Prevention, 2025a).

Our study is not without limitations. PCA does not consider the biological relevance of individual factors to BrainAGE/cognition, as components are derived purely from statistical variance within metabolism, independently of their association with any given outcome. Additionally, considering the complexity of treatment regimens and the noise inherent in quantifying past medication use, it is difficult to accurately capture the effects of medication exposure in a cross-sectional study. Interestingly, we did not detect an association between obesity-related brain changes and cognition. This likely relates to the sensitivity of BrainAGE to cognitive alterations, and the different sensitivity to detect brain and cognitive changes. Specifically, our brain measures are broad and not cognitive task-specific. Verbal memory-related cognitive changes may be associated with more localized alterations in brain structure and may not be captured by a broad measure such as BrainAGE (Richard et al., 2018) (Lee et al., 2022). Furthermore, our sensitivity to detect subtle cognitive changes is likely greater than our sensitivity to detect microscopic brain changes. In other words, BrainAGE may lack the sensitivity to capture the more subtle cognitive impairments observed in BD or MetSy. Nonetheless, this finding may suggest that associations between metabolic dysfunction and the brain or cognition may occur independently and in parallel to one another. Finally, as our study is cross-sectional, we cannot infer the temporal direction or the causal nature of these associations. Our mediation analyses reflect statistical decomposition of cross-sectional associations, not causal mediation.

To conclude, this study is the first to our knowledge to demonstrate in the same sample that MetSy statistically explains part of the association between BD and brain structure or cognition. Notably, over a quarter of brain/cognitive alterations associated with BD were explained by MetSy and mostly related to obesity. The negative effects of MetSy were independent of and additive to the negative effects of the diagnosis of BD and antipsychotic treatment. Our findings, therefore, suggest that poor metabolic health, especially obesity, is substantially associated with neurostructural and cognitive alterations in BD. While the need for metabolic monitoring should be informed simply by the diagnosis of BD, it is important to realize that screening for MetSy can also help track brain health and cognitive functioning. Future studies should test whether treatment of metabolic changes may at least partially reverse brain and cognitive alterations and improve overall functioning/quality of life in BD.

## Supporting information

10.1017/S0033291726104905.sm001 Selitser et al. supplementary material Selitser et al. supplementary material

## Supplementary material

The supplementary material for this article can be found at http://doi.org/10.1017/S0033291726104905.

## Table 1. Long description

From the top, columns are labeled Control, Patient, and Significance of Group Difference. The first row shows sample size: Control n equals 87, Patient n equals 76. Age is higher in patients, mean 46.82 years versus 39.88, F (1,159) equals 8.76, p equals 0.004 double asterisk. Sex percent male is similar: 39.1 percent in controls, 44.7 percent in patients, not significant. Diabetes percent yes is 9.8 in controls, 21.0 in patients, not significant. Hypertension percent yes is 14.9 in controls, 28.9 in patients, not significant. Overweight or obese, BMI greater than 25 kg per meter squared, is 55.2 percent in controls, 77.6 percent in patients, F (1,158) equals 5.78, p equals 0.017 single asterisk. Triglycerides, mmol per liter, mean 1.11 in controls, 1.61 in patients, F (1,150) equals 8.05, p equals 0.005 double asterisk. L D L, mmol per liter, is similar between groups. H D L, mmol per liter, is lower in patients, 1.26 versus 1.50, F (1,150) equals 16.05, p less than 0.001 triple asterisk. Fasting glucose, H b A 1 c, HOMA I R, and C R P show no significant differences. Systolic and diastolic blood pressure are higher in patients but not significant. BMI mean is 30.26 in patients, 26.12 in controls, F (1,158) equals 18.25, p less than 0.001 triple asterisk. Waist circumference and waist-to-hip ratio are higher in patients, both highly significant. All comparisons are adjusted for age and sex.

Navigate back to Table 1..

## Table 2. Long description

Starting from the top, columns are Control, Patient, and Significance of group difference. N is 87 for Control, 76 for Patient. For CVLT trial I, means are 7.24 (2.10) for Control, 6.61 (2.21) for Patient, F (1,120) equals 1.04, p equals 0.309. For CVLT trial V, Control is 13.52 (2.10), Patient is 12.07 (2.72), F (1,120) equals 6.13, p equals 0.015, marked with a single asterisk. Sum trials I-V: Control 54.77 (10.76), Patient 48.58 (11.43), F (1,158) equals 7.64, p equals 0.006, double asterisks. List B: Control 6.32 (2.08), Patient 5.14 (1.96), F (1,158) equals 8.36, p equals 0.004, double asterisks. Short delay free recall: Control 11.90 (3.00), Patient 9.97 (3.47), F (1,158) equals 8.34, p equals 0.004, double asterisks. Long delay free recall: Control 12.24 (3.13), Patient 10.51 (3.63), F (1,156) equals 6.44, p equals 0.012, single asterisk. Total intrusions: Control 2.06 (2.59), Patient 2.76 (3.50), F (1,158) equals 0.11, p equals 0.739. Total repetitions: Control 3.36 (3.26), Patient 2.38 (2.50), F (1,158) equals 2.75, p equals 0.099. Learning slope: Control 6.27 (1.96), Patient 5.46 (2.42), F (1,119) equals 5.09, p equals 0.026, single asterisk. Retroactive interference: Control minus 1.76 (1.87), Patient minus 1.80 (2.06), F (1,120) equals 0.12, p equals 0.725. Proactive interference: Control minus 0.65 (2.12), Patient minus 1.15 (2.22), F (1,120) equals 1.5, p equals 0.223. Forward digit span: Control 11.01 (2.27), Patient 11.10 (2.45), F (1,145) equals 0, p equals 0.960. Backward digit span: Control 9.25 (2.46), Patient 8.75 (2.36), F (1,145) equals 1.33, p equals 0.252. Significant group differences (p less than 0.05) are present for CVLT trial V, sum trials I-V, List B, short and long delay free recall, and learning slope. All comparisons are adjusted for age and sex, with learning slope also adjusted for CVLT trial I score.

Navigate back to Table 2..

## Figure 1. Long description

From the bottom left, the y-axis lists H D L, L D L, HOMA I R, B M I, Waist-to-Hip Ratio, Systolic B P, Diastolic B P, H b A 1 c, and Triglycerides. The x-axis repeats these variables in the same order. Each cell represents the correlation between the intersecting variables, colored from blue for negative correlations to red for positive correlations. The strongest positive correlations are seen between B M I and W H R, HOMA I R and B M I, and L D L and H D L, indicated by dark red. Negative correlations, such as H D L with Triglycerides and H D L with L D L, are shown in blue. The color scale on the right ranges from minus 1.0 (blue) to plus 1.0 (red), with intermediate values in lighter shades. Minus signs are not visually present but implied by blue coloration.

Navigate back to Figure 1..

## Figure 2. Long description

From left to right, panel A plots Metabolic P C 1 on the x axis and Brain AGE (adjusted) on the y axis, with a positive trend line and R equals 0.16, p equals 0.038. Panel B plots Metabolic P C 1 on the x axis and Cognitive P C 1 (adjusted) on the y axis, with a negative trend line and R equals minus 0.16, p equals 0.047. Panel C plots Brain AGE on the x axis and Cognitive P C 1 (adjusted) on the y axis, with a weak negative trend line and R equals minus 0.08, p equals 0.31. Each panel contains scattered black points representing individual data, and all axes are labeled accordingly.

Navigate back to Figure 2..

## Figure 3. Long description

Panel A at the top shows Brain AGE (adjusted) on the y-axis, with control and patient groups on the x-axis. The patient group has a higher mean Brain AG E, with non-overlapping 95 percent confidence intervals, marked by a single asterisk above the bracket. Panel B at the bottom left shows Metabolic P C 1 (adjusted) on the y-axis, with controls and patients on the x-axis. The patient group has a higher mean Metabolic P C 1 score, with non-overlapping 95 percent confidence intervals, indicated by three asterisks above the bracket. Panel C at the bottom right shows Cognitive P C 1 (adjusted) on the y-axis, with controls and patients on the x-axis. The patient group has a lower mean Cognitive P C 1 score, with non-overlapping 95 percent confidence intervals, indicated by two asterisks above the bracket. All panels use black dots for group means and vertical bars for 95 percent confidence intervals.

Navigate back to Figure 3..

## Figure 4. Long description

The diagram contains two panels. In the left panel, Bipolar Disorder is at the base, with arrows pointing to Metabolic P C 1 (top) and Brain AGE (right). The arrow from Bipolar Disorder to Metabolic P C 1 is labeled 1.25 (0.31), p less than 0.001, three asterisks. The arrow from Metabolic P C 1 to BrainAGE is labeled 0.85 (0.28), p equals 0.002, two asterisks. The direct arrow from Bipolar Disorder to BrainAGE is labeled 2.93 (1.13), p equals 0.01, one asterisk. A C M E equals 0.87 [0.15, 1.74], p equals 0.01, one asterisk, is above Metabolic P C 1, with 30 percent mediated. A D E equals 2.06 [-0.2462, 4.16], p equals 0.06 is below the direct path. In the right panel, Bipolar Disorder is at the base, with arrows to Metabolic P C 1 (top) and Cognitive P C 1 (right). The arrow from Bipolar Disorder to Metabolic P C 1 is labeled 1.01 (0.24), p less than 0.001, three asterisks. The arrow from Metabolic P C 1 to Cognitive P C 1 is labeled negative 0.244 (0.09), p equals 0.005, two asterisks. The direct arrow from Bipolar Disorder to Cognitive P C 1 is labeled negative 0.74 (0.27), p equals 0.007, two asterisks. A C M E equals negative 0.19 [-0.39, -0.03], p equals 0.01, one asterisk, is above Metabolic P C 1, with 25 percent mediated. A D E equals negative 0.56 [-1.14, -0.04], p equals 0.04, one asterisk, is below the direct path. All paths control for age, sex, and lithium treatment where applicable.

Navigate back to Figure 4..

## Table 3. Long description

The table has two main columns: Controls and Patients. From top to bottom, the rows are as follows. N: Controls 87, Patients 76. Age of onset: Controls NA, Patients 23.99 (9.30). BD type (Type 2 percent): Controls NA, Patients 21 (27.6). Number of prior episodes: Controls NA, Patients 10.35 (11.28). Number of prior hospitalizations: Controls NA, Patients 2.22 (4.94). Duration of illness in years: Controls NA, Patients 20.74 (12.00). ADHD (Yes percent): Controls 1 (1.1), Patients 2 (2.6). Anxiety disorders (Yes percent): Controls 6 (6.9), Patients 24 (31.6). Eating disorders (Yes percent): Controls 0 (0.0), Patients 1 (1.3). Substance use disorder (Yes percent): Controls 1 (1.1), Patients 12 (15.8). Other psychiatric disorder (Yes percent): Controls 1 (1.1), Patients 9 (11.8). Antipsychotics (Yes percent): Controls 0 (0.0), Patients 38 (50.0). Anticonvulsants (Yes percent): Controls 2 (2.3), Patients 36 (47.4). Antidepressants (Yes percent): Controls 2 (2.3), Patients 30 (39.5). Benzodiazepine (Yes percent): Controls 0 (0.0), Patients 15 (19.7). Lithium (Yes percent): Controls 0 (0.0), Patients 42 (55.3). HAM D score: Controls 1.25 (1.97), Patients 5.41 (5.44). Y M R S score: Controls 0.60 (1.25), Patients 1.92 (3.04).

Navigate back to Table 3..
