# Supplementary material for: The role of metabolic health in neurostructural and cognitive alterations in bipolar disorders
Source: Psychol Med. 2026 Jun 18;56:e197. doi: 10.1017/S0033291726104905 (PMC13280689; doi:10.1017/S0033291726104905)
Supplement: Selitser et al. supplementary material [file S0033291726104905sup001.docx]

**Supplementary Materials**

**Table S1** Loadings from the metabolic principal component analysis

|  | PC1 | PC2 | PC3 |
| --- | --- | --- | --- |
| Fasting HDL | -0.266510 | -0.234910 | -0.637150 |
| Fasting LDL | 0.148155 | -0.518450 | 0.015348 |
| HOMA-IR | 0.379274 | 0.330194 | -0.169250 |
| BMI | 0.425079 | 0.043511 | -0.063870 |
| WHR | 0.408106 | 0.084410 | 0.005373 |
| Systolic BP | 0.403137 | -0.296390 | -0.180090 |
| Diastolic BP | 0.338546 | -0.378600 | -0.317180 |
| HbA1c | 0.112111 | 0.568906 | -0.493930 |
| Fasting TGC | 0.355285 | 0.056023 | 0.429012 |

**Table S2** Loadings from the cognitive principal component analysis

|  | PC1 | PC2 | PC3 |
| --- | --- | --- | --- |
| Sum Trials I-V | 0.454930 | -0.033100 | -0.004490 |
| Short Delay Free Recall | 0.508066 | -0.114720 | 0.153538 |
| Long Delay Free Recall | 0.491053 | -0.042620 | 0.131007 |
| Total Intrusions | -0.234370 | -0.068550 | -0.182530 |
| Total Repetitions | 0.082529 | 0.102876 | -0.286630 |
| Learning Slope | 0.200344 | 0.613793 | 0.286954 |
| Retroactive Interference | 0.291079 | -0.461210 | 0.150735 |
| Proactive Interference | -0.045850 | 0.517687 | 0.370803 |
| Forward Digit Span | 0.184702 | 0.251878 | -0.583510 |
| Backward Digit Span | 0.266372 | 0.218509 | -0.510610 |

**Table S3** Effects of CRP on Metabolic PC1, BrainAGE, Cognitive PC1

| **Predictor** | **Effect on Metabolic PC1** |
| --- | --- |
| Age | *β* = 0.03 (±0.01), *p <* 0.001*** |
| Sex (Male) | *β* = 1.32 (±0.23), *p < 0.001**** |
| CRP | *β* = 0.61 (±0.09), *p <* 0.001***** |
| **Predictor** | **Effect on BrainAGE** |
| Age | *β* = -0.28 (±0.03), *p <* 0.001*** |
| Sex (Male) | *β* = 1.17 (±0.97), *p = 0.235* |
| CRP | *β* = 0.36 (±0.40), *p* = 0.368 |
| **Predictor** | **Effect on Cognitive PC1** |
| Age | *β* = -0.03 (±0.009), *p <* 0.001*** |
| Sex (Male) | *β* = -0.78 (±0.29), *p < 0.01*** |
| CRP | *β* = 0.04 (±0.12), *p =* 0.716 |
|  |  |

**Table S4** Effects of clinical variables and Metabolic PC1 on BrainAGE among BD patients

| **Predictor** | **Effect on BrainAGE** |
| --- | --- |
| Age | *β* = -0.30 (±0.04), *p <* 0.001*** |
| Sex (Male) | *β* = 0.90 (±1.09), *p =* 0.41 |
| Metabolic PC1 | *β* = 1.03 (±0.35), *p <* 0.01** |
| Antipsychotics | *β* = 2.56 (±1.08), *p =* 0.02*** |
| Anticonvulsants | *β* = 1.49 (±1.09, *p =* 0.18 |

**Table S5** Effects of clinical variables and Metabolic PC1 on Cognitive PC1 among patients.

| **Predictor** | **Effect on BrainAGE** |
| --- | --- |
| Age | *β* = 0.004 (±0.02), *p =* 0.83 |
| Sex (Male) | *β* = -1.00 (±0.41), *p =* 0.02* |
| Metabolic PC1 | *β* = -0.10 (±0.13), *p =* 0.47 |
| Antipsychotics | *β* = -1.05 (±0.40), *p =* 0.01*** |
| Duration of Illness | *β* = -0.06 (±0.02), *p =* 0.03* |

|  |  |
| --- | --- |
|  |  |
|  |  |
